# The Advances of Single-Cell RNA-Seq in Kidney Immunology

**DOI:** 10.3389/fphys.2021.752679

**Published:** 2021-10-13

**Authors:** Honghui Zeng, Xiaoqiang Yang, Siweier Luo, Yiming Zhou

**Affiliations:** ^1^Guangdong Provincial Key Laboratory of Malignant Tumor Epigenetics and Gene Regulation, Guangdong-Hong Kong Joint Laboratory for RNA Medicine, Sun Yat-sen Memorial Hospital, Sun Yat-sen University, Guangzhou, China; ^2^Medical Research Center, Sun Yat-sen Memorial Hospital, Sun Yat-sen University, Guangzhou, China

**Keywords:** kidney, immune system, single-cell RNA sequencing, lupus nephritis, diabetic kidney disease, IgA nephropathy, anti-neutrophil cytoplasmic antibody-associated glomerulonephritis

## Abstract

Kidney diseases are highly prevalent and treatment is costly. Immune cells play important roles in kidney diseases; however, it has been challenging to investigate the contribution of each cell type in kidney pathophysiology. Recently, the development of single-cell sequencing technology has allowed the extensive study of immune cells in blood, secondary lymphoid tissues, kidney biopsy and urine samples, helping researchers generate a comprehensive immune cell atlas for various kidney diseases. Here, we discuss several recent studies using scRNA-seq technology to explore the immune-related kidney diseases, including lupus nephritis, diabetic kidney disease, IgA nephropathy, and anti-neutrophil cytoplasmic antibody-associated glomerulonephritis. Application of scRNA-seq successfully defined the transcriptome profiles of resident and infiltrating immune cells, as well as the intracellular communication networks between immune and adjacent cells. In addition, the discovery of similar immune cells in blood and urine suggests the possibility of examining kidney immunity without biopsy. In conclusion, these immune cell atlases will increase our understanding of kidney immunology and contribute to novel therapeutics for patients with kidney diseases.

## Background

The kidneys are vital organs serving critical functions in the human body, including clearance of waste from blood, maintenance of the salt/water balance, and regulation of blood pressure. In addition to these functions, the kidneys also maintain the homeostasis of the immune system through filtration and excretion of bacterial toxins, circulating cytokines, and inflammatory molecules (Kurts et al., 2013; Donnan et al., 2021). Although considerable progress has been achieved in elucidating the contribution of immune cells in kidney diseases and in translating these results from the bench to the bedside, determining how these immune cells orchestrate kidney immunology in health and disease remains a challenge due to the relatively small number of immune cells and the highly complex cell composition of kidneys. Hence, our understanding of kidney immunology in humans remains incomplete.

In health, the major kidney resident immune cells are dendritic cells (DCs) and macrophages (Cao et al., 2011; Kurts et al., 2013, 2020). A small number of lymphocytes are also present in healthy kidneys (Turner et al., 2018). Therefore, the kidneys, similar to the spleen, can maintain the peripheral tolerance through the kidney resident antigen-presenting cells (APCs). This becomes more obvious in end-stage renal disease (ESRD), where both kidney function and the immune system are severely compromised. Retention of excessive toxins and cytokines in ESRD activates innate immune cells and increases the production of cytokines and proinflammatory molecules, which further causes kidney damage (Betjes, 2013). Kidney-resident DCs, derived from common DC precursors (CDPs) and monocytes, are located in the tubulointerstitium and absent in the glomeruli (Teteris et al., 2011). Resident DCs in the kidney appear to be crucial for maintaining peripheral tolerance via direct interaction with filtered antigens in the tubular lumen regions and for presenting these antigens to T cells in lymph nodes, which may be more efficient in kidneys than in other organs. In ischemia-reperfusion (IR) and unilateral ureter obstruction (UUO) mouse models, resident DCs promoted kidney injury by producing proinflammatory cytokines. In contrast to DCs, kidney-resident macrophages are scattered in the renal cortex and infiltrate into the tubulointerstitium during tissue damage. Some evidence suggests that kidney-resident macrophages play protective roles during acute and chronic kidney diseases. Studies found that the lack of resident kidney CD45^+^ Ly6G-F4/80^high^CD11b^int^ macrophages, which express an inhibitory immune checkpoint molecule, V-domain Ig suppressor of T cell activation (VISTA), delays tissue repair in several animal models (Park et al., 2020). CD11b^int^F4/80^bright^ kidney resident macrophages were found to be protective in the ischemic kidney by promoting proangiogenic environments (Puranik et al., 2018). In contrast, other studies argue that activation of macrophages aggravates kidney injury through the production of proinflammatory cytokines (Yang et al., 1998; Isbel et al., 2001; Eardley et al., 2008). Thus, kidney-resident macrophages seem to have diverse effects depending on cell identity, the kidney injury type and the disease stage.

On one hand, kidneys can be the direct target of the immune system, by which lymphocytes and/or antibodies interact with kidney cells and lead to kidney damage. Tubulointerstitium DCs might capture glomerular antigens, which in turn induce infiltrating T cells to produce proinflammatory cytokines. Infiltrating monocytes also contribute to local inflammation and tissue injury, which have been shown to cause tubular atrophy and interstitial scarring. On the other hand, kidneys can be an indirect victim of immune system dysregulation, such as the immune complexes deposition in glomeruli that promote local inflammation. Immune complexes can be a result of either an immune response to infection or a systemic autoimmune disease condition (e.g., SLE). In both cases, activation of innate and adaptive immune cells, as well as the complement system, play important roles in various kidney diseases.

Although a number of previous studies have confirmed the contribution of immune cells in kidney diseases (Kurts et al., 2013; Donnan et al., 2021), each of these studies focus on fewer than three immune cell populations. To date, therefore, our knowledge and understanding of kidney immunology as a whole is still incomplete. Here, we discuss the recent progress and findings in kidney diseases uncovered by scRNA-seq technology. We briefly introduce the general principles and current methodologies of scRNA-seq as well as the experimental design for basic and clinical research related to the kidney field. We then discuss recent findings focusing on several immune-related kidney diseases, including LN, DKD, IgAN, and ANCA-GN, highlighting the changes in immune cell populations and the possible immune mechanisms revealed by scRNA-seq technology. Finally, we discuss how the generation of immune cell atlases might help transform these results into immunological therapies in the future.

## Comparison of Single-Cell RNA-Seq Methods

**FIGURE 1:**
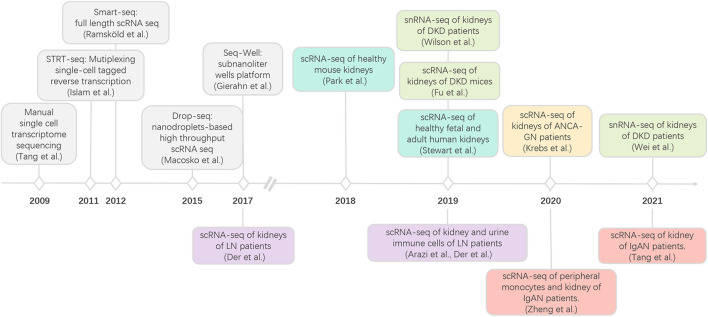
Development of scRNA-seq and its applications in kidney immunology. LN, lupus nephritis; diabetic kidney disease; IgAN, IgA nephropathy; ANCA-GN anti-neutrophil cytoplasmic antibody-associated glomerulonephritis.

scRNA-seq technology has been rapidly developed in recent decades (Figure 1; Tang et al., 2009; Islam et al., 2011; Ramskold et al., 2012; Macosko et al., 2015; Gierahn et al., 2017), enabling the characterization of transcriptome profiles in heterogeneous cell populations at the single-cell resolution (Wagner et al., 2016). To date, several scRNA-seq methods are commercially available; each with distinct strengths and limitations with respect to experiment throughput, detection sensitivity, gene coverage, and cost per cell (Table 1). General workflow of scRNA-seq includes single-cell preparation, cell capture and lysis, mRNA capture and reverse transcription, cDNA amplification, library construction, next-generation sequencing, and computational analysis. According to the sequencing length, single-cell RNA-seq can be divided into full-length, 3′ and 5′ end sequencing methods. Full-length sequencing captures the whole mRNA sequences and has a deeper sequencing depth, which make it very useful in studying genes related to alternative splicing or at low levels. Both 3′ and 5′ end sequencing has higher experimental throughput and lower cost per cell. Currently, 3′ end sequencing is more frequently used for whole transcriptomic assays, while the 5′ end is used to detect the clonal diversity of T and B cells trough TCR or BCR V(D)J sequencing (Papalexi and Satija, 2018). With the rapid development and commercialization of these scRNA-seq methods, an increasing number of open-source computational tools, from data quality control, batch effect correction, dimension reduction analysis, to data visualization, have been developed, which further made scRNA-seq technology applicable to most researchers. To date, several scRNA-seq methods have been used to investigate more than 10,000 cells simultaneously, allowing us to identify both rare and novel cell populations, investigate the transcriptomic changes in each cell and analyze the intercellular communication networks. As mentioned above, single-cell preparation is the first step of all scRNA-seq technologies which require fresh tissue samples and successful dissociation of tissue to generate good quality data and cells should be smaller than the droplets or microwells. To overcome these technical barriers, researchers developed the single-nucleus RNA-sequencing (snRNA-seq) method (Krishnaswami et al., 2016; Lacar et al., 2016; Habib et al., 2017), where nuclear, not cytoplasmic RNA is sequenced. Although the RNA-splicing information is lost and the detection sensitivity is reduced because of the increased level of pre-mRNA, snRNA-seq is particularly helpful in studying the dissociation-resistant or frozen samples, and cells with diameters over 50 μm. Therefore, researchers should carefully select scRNA-seq methods for their studies based on the tissue origin, sample size, sample storage method, and target cell abundance. It is highly recommended that target cells should be enriched by FACS when their population is less than 5% of the total.

**TABLE 1:** List of scRNA-seq methods.

	**SMART-seq/C1**	**SMART-seq2**	**CEL-seq2/C1**	**10× Genomics chromium**	**BD rhapsody**
Single cell preparation and capture	Fluidigm C1 platform	FACS	C1 platform	10**×** Genomics chromium platform	BD rhapsody platform
Sample preparation	Complicated	Complicated	Complicated	Easy	Easy
Quality check	Microscope	No	Microscope	No	BD rhapsody platform
UMI-based	No	No	Yes	Yes	Yes
cDNA coverage	Full-length	Full-length	3′ counting	5′ or 3′ counting	5′ or 3′ counting
Amplification method	TS-based PCR	TS-based PCR	*In vitro* transcription	TS-based PCR	TS-based PCR
Sample multiplexing	No	No	No	Yes	Yes
Cell number limitation	5–10, 10–17, or 17–25 μm	Less than 50 μm	Less than 50 μm	Less than 50 μm	Less than 50 μm
Required cell numbers per run	>10,000	No limitation	>10,000	>10,000	>20,000
Long term storage	No	Yes	No	No	Yes
Throughput capability	Limited by number of machines	Limited by operator efficiency	Limited by number of machines	Up to 8 samples per chip	Limited by operator efficiency
Cost	High	High	Medium	Low	Low

A study showed that snRNA-seq performed well on inflamed fibrotic tissue and could capture more cell types including glomerular podocytes, mesangial cells, endothelial cells, and juxtaglomerular cells with reduced dissociation-induced bias and transcriptional stress responses compared to scRNA-seq (Wu et al., 2019).

In addition to aforementioned scRNA-seq methods, which provide robust information of different cell populations but lose the spatial characteristics of samples, high-throughput spatial transcriptomics techniques were developed and most of them recorded the spatial information by labeling mRNA transcripts with location barcodes. Following detachment and deep sequencing, spatial transcriptomics techniques align transcripts to their capture spots of origin based on their location barcode (Longo et al., 2021). Spatial transcriptomics techniques were powerful in resolving the dynamic change within the niches under specific biological context, and efforts were made to increase the resolution and integrated the scRNA seq with spatial transcriptomes.

In most cases, scRNA-seq was employed *in vivo* experiment but its application *in vitro* also helps people to resolve complex kidney pathogenic mechanism, especially when studying cells underwent asynchronous differentiation or continuous transition. Kidney organoids which were generated from human pluripotent stem cells had self-organizing 3D structure and mimicked the functions of human kidney. Kidney organoids emerged as human-based models to study multiple kidney cells as a whole *in vitro* and serve to biomarker discovery and drug invention (Bonventre, 2018). By performing scRNA-seq of kidney organoids, Digby et al. (2020) found that kidney organoid demonstrated phenotype of acute kidney injury (AKI) with higher expression of KIM1 and increased cell damage. The main injured cell types of cisplatin was change as concentration altered with interstitial cells at high dose (50 μM) and proximal tubule cell at low repeated dose. Their study validates the use of kidney organoids to model AKI *in vitro* and provided a suitable stimulation protocol to study AKI. Macrophage-myofibroblast transition (MMT) is a newly discovered process and promote kidney fibrosis in a TGF-β/Smad3-dependent pattern (Meng et al., 2016; Wang et al., 2016). By performing scRNA-seq to study sorted TGF-β1-treated Smad3^+/+^ and Smad3^–/–^ bone marrow-derived macrophages (BMDMs), Tang et al. (2018) uncovered that the proto-oncogene Src is a direct target gene of Smad3 and serves as a centric hub in the gene regulatory network during MMT. They also found that neural transcription factor Pou4f1 was predicted as the only transcription factor involved in MMT, and later experiments validated that Pou4f1 promoted MMT-mediated fibrosis via a fibrogenic gene network including Fn1, Pdgfrb, Itgb3 et al. (Tang et al., 2020).

## Generation of a Kidney Cell Atlas Through scRNA-seq

Recently, a number of studies have been published using scRNA-seq technology in kidney research, from those seeking to discover rare and novel kidney cell types to elucidating cell type-specific contributions to kidney diseases and understanding intracellular communication networks (Table 2). A groundbreaking progress is the generation of healthy kidney cell atlas, which can serve as a reference transcriptomic map for the future studies (Park et al., 2018). Park et al. (2018) applied scRNA-seq to healthy mouse kidneys and provided a molecular definition of kidney cells. They found five clusters of immune cells in the kidney including macrophages, neutrophils, natural killer cells, B lymphocytes and T lymphocytes. Among them, T lymphocytes had the largest number. They also discovered a novel collecting duct cell population, which expresses both intercalated and principal cell markers. They found that a transition between cells of this subpopulation. When compared with the transcriptional profiles of kidney cells from a mouse fibrosis model, these novel cells discovered by Park et al. (2018) exhibited a shift in the transition balance toward principal cells. Since intercalated cells play critical roles in proton secretion, a decreased number of intercalated cells may lead to metabolic acidosis in CKD patients.

**TABLE 2:** Summary of scRNA-seq in kidney diseases.

**Author information**	**Arazi et al., 2019**	**Der et al., 2019**	**Fu et al., 2019**	**Wilson et al., 2019**	**Zheng et al., 2020**	**Tang et al., 2021**	**Krebs et al., 2020**
Disease type	LN	LN	DKD	DKD	IgAN	IgAN	ANCA-GN
scRNA-seq method	Modified CEL-Seq2	Fluidigm C1	Fluidigm C1	10× chromium	Modified STRT-seq	Singleron Matrix	10× chromium CITE-seq
Species	Human	Human	Mouse	Human	Human	Human	Human and mouse
Tissue origin	Kidney biopsy, blood	Kidney and skin biopsy, blood	Kidney tissue	Kidney biopsy, blood	Kidney biopsy, blood	Kidney biopsy, blood	Kidney biopsy, blood
Human sample number	10 control	3 control	3 control	3 non-diabetic	5 control	1 control (GSE131685)	3 control
	24 LN	21 LN kidney biopsy, 17 skin biopsy	3 STZ-diabetes	3 Diabetes	5 IgAN	4 IgAN	3 ANCA-GN
Cell number for analysis	2,736	4,019	829	23,980	8,880	20,570	5,905
Features of the study	21 leukocyte clusters; local activation of B cells; renal differentiation of monocytes; type 1 interferon response genes in most cells; broad expression of CXCR4 and CX3CR1	Enriched type I interferon response genes; association of high IFN response and fibrotic genes in tubular cells with non-responders	Increased number of immune cells in DKD glomeruli; macrophages are major immune cell type; higher number of M1 macrophages than M2	4 clusters of immune cells, including T, B, monocytes, and plasma cells; increased number of leukocytes; increased expression of TNFRSF21 in monocytes	Increased interactions between mesangial cells, macrophages, and T cells; increased Notch, glycolysis, fatty acid, and amino-acid metabolism genes in macrophages; increased expression of CCL2 and CX3CR1 in macrophages	Detected macrophages, monocytes, and DCs but not T cells in IgAN kidney; decreased expression of GPX3, FAM49B, and FCGBP in macrophages; increased expression of CCL2 and CXCL1; increased expression of Notch, FGF2 and PDGFD in mesangial cells	12 clusters of renal T cells; CCR6^high^CCR7-CD69^+^ CD4^+^ T_RM_ cells display Th17 signature in ANCA-GN; pathogen infection induces T_RM_17 cells in kidney; increased level of IL-17A from T_RM_17 cells

To define the heterogeneity among epithelial, myeloid, and lymphoid cells, Stewart et al. (2019) performed scRNA-seq using healthy fetal and adult kidney samples. They discovered that several immune cell populations in fetal kidneys appear at the different time points. Certain types of DCs and macrophages were found to be present at the earliest development stage, while NK cells, T cells, and monocytes appeared at 9 weeks of gestation, and B cells were found to present at 12 weeks. These data reveal the temporal development of immune cells at single-cell resolution in human fetal kidney. Using mature kidney samples, this group identified a higher number of immune cells, including resident macrophages, pDCs, neutrophils, mast cells, T, B, NK, and NKT cells, compared to that of fetal kidney samples. In the lymphoid compartment of fetal kidney, B cells did not express class-switching genes, and CD8 + T cells expressed the low effector gene GZMH, while in healthy mature kidneys, B cell clusters expressed IgM, IgG, and IgA, and polarization of CD4 + T cells was not found through an analysis of cytokine and transcription factor expression. Within the NK clusters, there were cells expressing both γ- and δ-T cell receptors as well as markers of mucosal-associated invariant T (MAIT) cells. They also demonstrated that CD8 + T and NK cells in fetal kidneys show decreased enrichment with genes involved in “T cell receptor signaling” and “NK cell-mediated immunity,” respectively, compared to those cells in mature kidneys. Interestingly, Stewart et al. (2019) also discovered that the transcriptome profiles of macrophages in both fetal and mature kidneys resembled those of anti-inflammatory M2 macrophages. Compared with the monocytes in fetal kidney, monocyte-derived macrophages displayed higher levels of phagocytosis genes and defense genes to bacteria in mature kidney. Taken together, these studies provide a novel immune cell landscape in mouse and human kidneys, which can be used as a reference dataset and will facilitate the future study of pathogenic mechanisms.

## scRNA-Seq in Lupus Nephritis

Lupus nephritis (LN) is caused by systemic lupus erythematosus (SLE), an autoimmune disorder in which the immune system targets the body’s own cells (Davidson and Aranow, 2010; Tsokos, 2011). LN occurs when lupus autoantibodies attack the kidneys, which leads to hematuria, proteinuria, impaired kidney function, and even kidney failure. Considerable evidence from pathological and bulk RNA-seq analyses of LN kidney samples suggests that the infiltration of lymphocytes is closely associated with reduced kidney function as well as a poor patient prognosis. One early study, in which scRNA-seq was performed with kidney and skin samples from patients with LN and with skin samples from healthy donors, generated data on 899 cells (Der et al., 2017). This study demonstrated a correlation between type I interferon signaling in renal tubular cells and skin keratinocytes in patients with active lupus nephritis, suggesting the potential of evaluating LN activity with skin biopsy samples. Unfortunately, the immune cells identified in this study were very limited due to the small number of sequenced cells, although some T cells and myeloid cells were observed in patients with LN.

Later, using strategies to enrich immune cells before sequencing, two studies reported detailed immune cell landscapes of the kidneys in LN patients (Arazi et al., 2019; Der et al., 2019). Both studies suggested that an abundant number of immune cells are present in the kidneys of patients with LN, including inflammatory and phagocytic macrophages, DCs, NK cells, B cells, and a group of memory T cells. The infiltrating myeloid and lymphoid cells detected by scRNA-seq were confirmed using immunohistochemical and immunofluorescent methods. Interestingly, a subset consisting of B and plasma cells, which are the major sources of autoantibodies, was found to express type I interferon signature genes in LN kidneys, implying that these cells play important roles in LN pathogenesis. In normal kidney tissue, there are two predominant immune cell populations: a population of myeloid cells that differs from the blood myeloid cell population and a population of effector memory CD4 + T cells. Notably, with scRNA-seq, no B cells were found in healthy kidneys, which was confirmed by the flow cytometry assay. These results identify the cell populations that contribute to immune-mediated kidney injury in lupus nephritis and provide novel insights into B cells and plasma cells, showing that they may contribute to LN pathogenesis through clonal expansion at the site of injury; however, further studies are needed to investigate the detailed mechanism.

In addition, Arazi et al. (2019) demonstrated the possibility of performing scRNA-seq with immune cells in urine samples, which provides a non-invasive way to study immune cells in kidneys. Previously, Park et al. (2018) mapped the disease-associated genes, which were identified from the GWAS and other genetic studies, to their scRNA-seq dataset of mouse kidneys and generated a cell type-specific expression profile of these disease-associated genes. Similar to this approach, the Accelerating Medicines Partnership Network integrated risk genes from GWAS of SLE patients with the scRNA-seq data generated from patients with LN (Der et al., 2017; Arazi et al., 2019). Therefore, this approach, the integration of scRNA-seq data and genetic data, provides additional information to understand the cell type-specific contribution to kidney disease pathogenesis.

## scRNA-Seq in Diabetic Kidney Disease

Diabetic kidney disease (DKD) is one of the most common microvascular complications of diabetic mellitus and a leading cause of end-stage renal disease (ESRD) worldwide (Reidy et al., 2014; Thomas et al., 2015). Although numerous studies have indicated that immune cells play essential roles in DKD pathogenesis, the detailed mechanism remains unelucidated (Wada and Makino, 2016; Flyvbjerg, 2017; Tang and Yiu, 2020). In a recent study, scRNA-seq was performed using DKD and normal kidney tissues, and showed that several types of immune cells are increased in the glomeruli of diabetic mice (Fu et al., 2019). The major immune cells detected were macrophages with highly expressed C1qa, Cd74, and Adgre1. Macrophages have been previously classified into two subtypes: M1 and M2 macrophages, which are associated with tissue damage and repair, respectively. Studies have demonstrated that the increased ratio of M1/M2 macrophages in kidneys strongly correlates with the urine albumin level and renal fibrosis in DKD (You et al., 2013). Fu et al. (2019) investigated macrophage clusters in DKD kidney samples based on 57 and 33 marker genes of M1 and M2, respectively. As expected, they found a higher number of M1 macrophages than M2 macrophages in the DKD samples. This result is consistent with previous studies showing that inflammatory macrophages are the major immune cells in the glomeruli of DKD. In addition, only a small number of other immune cells, such as neutrophils and B cells, were observed in the DKD kidney samples. Thus, this study suggests that dysregulation of the M1/M2 macrophage balance may contribute to tissue injury in DKD kidneys.

In contrast to Fu et al. (2019) study, Wilson et al. (2019) performed snRNA-seq and observed an approximate 7 fold increase in leukocyte number in diabetic patients; however, they failed to detect a significant number of resident macrophages in the diabetic samples. This discrepancy between two studies may be due to a limited sequencing cell number or different sequencing methods. Intriguingly, Wilson et al. (2019) found that infiltrating monocytes expressed the IFN gamma (IFNGR1 and IFNGR2) downstream signaling genes, such as HLA class II genes (HLA-DRB1, HLA-DRB5, HLA-DQA1), and TNFRSF1B, which are implicated as biomarkers for DKD (Benjafield et al., 2001; Robson et al., 2018; Xu et al., 2018). When comparing diabetic samples with 2 public datasets of PBMCs, Wilson et al. (2019) observed increased expression of TNFRSF21, one of the kidney risk inflammatory signature (KRIS) genes, in infiltrating CD14 + monocyte populations in diabetic samples. Interestingly, TNFRSF21 is also one of the few KRIS urinary markers that was correlated with enhanced urinary excretion and ESRD. Taken together, these studies indicate that macrophages/monocytes play important roles in DKD pathogenesis.

Using the snRNA-seq dataset generated by Wilson et al. (2019), Wei et al. (2021) found that DKD samples with high interstitial fibrosis and tubular atrophy displayed a higher cell number of B cells, suggesting that B cells may also play roles in DKD. Although a number of studies have confirmed the contribution of B cells in the pathogenesis of LN, recurrent focal segmental glomerulosclerosis, and membranous nephropathy (Fornoni et al., 2011; Gregersen and Jayne, 2012; Fervenza et al., 2019), the roles of B cells in DKD remains unclear. Studies have shown that the peripheral CD19^+^ CD38^+^ B cells was increased in diabetic patients (Smith et al., 2017). Notably, the number of CD19^+^ CD38^+^ B cells was found to be closely correlated with 24 h proteinuria level and was reduced after treatment. Taken together, these studies provide a landscape of immune cells in DKD and potential targets for DKD diagnosis and treatment.

## scRNA-Seq in IgA Nephropathy

IgA nephropathy (IgAN) is a common primary glomerulonephritis worldwide. Although this disease was initially described five decades ago, there are no specific or effective treatments to date. Approximately one-third of IgAN patients will progress to ESRD within 30 years after biopsy-based diagnosis (Roberts, 2014; Lai et al., 2016; Rodrigues et al., 2017). Recently, a “four-hit” model was proposed to explain the pathogenesis of IgAN: Unknown upstream factors cause the synthesis of galactose-deficient (gd)-IgA1 and the formation of gd-IgA-IgG immune complexes; deposition of immune complexes causes mesangial cell proliferation and secretion of inflammatory molecules and extracellular matrix (ECM) components; these molecules eventually lead to local activation of the immune system, glomerular sclerosis, mesangial expansion, and interstitial fibrosis (Lai, 2012; Wyatt and Julian, 2013; Roberts, 2014; Soares and Roberts, 2018). However, studies have shown that deposition of gd-IgA1-IgG immune complexes alone in the mesangium is not sufficient to induce severe kidney injury, suggesting that the accompanying inflammation contributes to IgAN pathogenesis, although the detailed mechanisms remain unclear.

To study circulating and resident immune cells, as well as other kidney cells, in IgAN, Zheng et al. (2020) applied a modified STRT-seq method and generated a single-cell transcriptome atlas of mesangial cells, epithelial cells, and circulating and resident immune cells. They applied the stepwise isolation method and captured major cell types in kidney cortex, generating a dataset with more than 3,000 genes and 30,000 transcripts per cell. Interestingly, they uncovered that mesangial cell of IgAN patients expressed higher levels of JCHAIN, a gene related to the dimerization and transportation of IgA molecules, which is predominately expressed in B cells. The increased expression level of this gene in mesangial cells may explain the mesangium-specific deposition of IgA immune complexes. This group also validated this result at the protein level; however, the detailed mechanism remains to be further investigated. In addition, Zheng et al. (2020) found that mesangial cells of IgAN patients expressed several inflammatory and ECM genes. In normal adult kidneys, mesangial cells are thought to clear immunoglobulins and ECM components; therefore, mesangial cell proliferation and ECM accumulation are tightly regulated (Mene et al., 1989). Under IgAN conditions, however, mesangial cells may have impaired functions in production and clearance of specific cellular components, leading to progressive kidney injury.

In another scRNA-seq study, Tang et al. (2021) discovered that mesangial cells of IgAN patients displayed the increased expression of MALAT1, GADD45B, SOX4, and EDIL3, which are also related to cell proliferation and matrix accumulation. The JCHAIN gene discovered by Zheng et al. (2020), however, did not appear in Tang et al. (2021) analysis results. This discrepancy might be due to the different sample dissociation methods (stepwise isolation vs. the GEXSCOPE tissue dissociation method) or the different sequencing platforms (modified STRT-seq vs. Singleron Biotechnologies platform). Nonetheless, both studies showed that mesangial cell proliferation and matrix accumulation play an essential role in IgAN pathogenesis.

It has been reported that the infiltration and accumulation of immune cells, especially macrophages/monocytes, in IgAN kidney tissue are associated with proteinuria and kidney damage in IgAN. However, the detailed mechanism of macrophage/monocyte recruitment remains unclear. Both studies discovered increased numbers of macrophages, monocytes, and DCs in the kidney of IgAN patients. Zheng et al. (2020) revealed that PLGRKT and CCL2, two cytokines that can recruit macrophages/monocytes, were highly expressed in mesangial cells of IgAN patients. Tang et al. (2021) found that three genes GPX3, FAM49B, and FCGBP, which are related to the mitochondrial function, ROS production, and EMT, respectively, were decreased in macrophages of IgAN. Taken together, these results provide new evidence showing how macrophages/monocytes contribute to IgAN pathogenesis. Furthermore, Zheng et al. (2020) found that the expression of effector T cell marker genes and cytotoxicity genes was significantly reduced, while the expression of T cell exhaustion genes was upregulated in CD8 + T cells of IgAN patients. These findings suggest a close association between CD8 + T cell dysfunction and IgAN pathogenesis; however, the detailed mechanism needs to be further investigated.

## scRNA-Seq in Anti-Neutrophil Cytoplasmic Antibody-Associated Glomerulonephritis

Anti-neutrophil cytoplasmic antibody (ANCA)-associated glomerulonephritis (ANCA-GN) is characterized by an autoimmune response to ANCAs and distinct glomerular lesions (Berden et al., 2010). Previous studies have indicated that distinct HLA class II haplotypes and CD4^+^ T cells are strongly associated with ANCA-GN (Spencer et al., 1992; Chanouzas et al., 2015). Substantial infiltrating CD4^+^ T cells was found in kidney biopsy samples of patients with crescentic GN. In addition, CD4^+^ T cells react to self-antigens in the active disease stage (Krebs et al., 2017). Experiments with rodent crescentic GN models suggested that CD4^+^ T cells, particularly Th17 cells, promote GN disease progression through the production of related cytokines, such as IL-17A, IL-17F, and IFN-γ. However, the contribution of other T cells to ANCA-GN pathogenesis remains unclear.

Therefore, Krebs et al. (2020) investigated T cells in ANCA-GN kidneys using scRNA-seq. Compared with healthy kidney samples, the authors found that samples of patients with ANCA-GN displayed a significantly increased number of CD4^+^ tissue resident memory T (T_RM_) cells. In addition, they discovered that a higher number of kidney resident CD69^+^ cells is negatively associated with kidney function in patients with active ANCA-GN. Interestingly, major subsets of CD4^+^ CD69^+^ T_RM_ cells exhibit transcriptomic profiles similar to that of Th1 or Th17 cells. They also displayed increased expression level of genes involved in cell proliferation, activation, and cytokine signaling. Therefore, CD4^+^ T_RM_ cells induced by pathogens may play an important role in aggravating ANCA-GN, although the detailed mechanism remains to be elucidated.

## Future Prospect

scRNA-seq methods have broadly expand our knowledge of kidney immunity and become more and more powerful and indispensable tools to study kidney diseases nowadays. Using scRNA-seq, people could detect gene expression alterations of specific cell clusters and distinguish universal and unique regulation pattern. scRNA-seq technologies not only help people to uncover mechanism under specific pathology process, but also offer useful information in the disease diagnosis. The diagnosis of many kidney diseases rely largely on invasive biopsy and histologic report. As mentioned before, Arazi et al. (2019) showed that the scRNA-seq profile of immune cells in urine samples was highly correlated. It is possible that scRNA-seq could be considered as a substitution of kidney biopsy as it becomes more economic in the future.

## Conclusion

scRNA-seq data generated in different laboratories on the basis of many kinds of human tissues will contribute to the Human Cell Atlas, an international database with a comprehensive and systematic reference map of human cells in health and disease. Eventually, this database will facilitate research by providing a reference transcriptome atlas at single-cell resolution. Currently, single-cell technology is increasing our understanding of kidney immunology at a revolutionary speed. It helps researchers understand cell heterogeneity, gene regulation, and cell-cell communication in kidney diseases, which will benefit disease diagnosis, therapeutic target identification, and off-target effect improvement. The studies discussed here applied single-cell technology to a wide range of immune-related kidney diseases using renal biopsy samples, cells in urine, and/or blood samples. scRNA-seq has led to the discovery of novel immune cell populations, gene regulation, and signaling pathways in immune-related kidney diseases. These findings will improve our understanding of kidney immunology in healthy individuals and patients with disease.

## Author Contributions

HZ, XY, SL, and YZ wrote the manuscript. All authors contributed to the article and approved the submitted version.

## Conflict of Interest

The authors declare that the research was conducted in the absence of any commercial or financial relationships that could be construed as a potential conflict of interest.

## Publisher’s Note

All claims expressed in this article are solely those of the authors and do not necessarily represent those of their affiliated organizations, or those of the publisher, the editors and the reviewers. Any product that may be evaluated in this article, or claim that may be made by its manufacturer, is not guaranteed or endorsed by the publisher.
